# Role of Long Non-coding RNAs on Bladder Cancer

**DOI:** 10.3389/fcell.2021.672679

**Published:** 2021-08-04

**Authors:** Hui-Jin Li, Xue Gong, Zheng-Kun Li, Wei Qin, Chun-Xia He, Lu Xing, Xin Zhou, Dong Zhao, Hui-Ling Cao

**Affiliations:** ^1^Shaanxi Key Laboratory of Ischemic Cardiovascular Disease, and Brain Disorders, Institute of Basic and Translational Medicine, Xi’an Medical University, Xi’an, China; ^2^College of Medical Technology, Xi’an Medical University, Xi’an, China

**Keywords:** bladder cancer (BC), long non-coding RNAs (lncRNAs), biomarker, early diagnosis, tumor development

## Abstract

Bladder cancer (BC) is the most common malignant tumor in the urinary system, and its early diagnosis is conducive to improving clinical prognosis and prolonging overall survival time. However, few biomarkers with high sensitivity and specificity are used as diagnostic markers for BC. Multiple long non-coding RNAs (lncRNAs) are abnormally expressed in BC, and play key roles in tumorigenesis, progression and prognosis of BC. In this review, we summarize the expression, function, molecular mechanisms and the clinical significance of lncRNAs on bladder cancer. There are more than 100 dysregulated lncRNAs in BC, which are involved in the regulation of proliferation, cell cycle, apoptosis, migration, invasion, metabolism and drug resistance of BC. Meanwhile, the molecular mechanisms of lncRNAs in BC was explored, including lncRNAs interacting with DNA, RNA and proteins. Additionally, the abnormal expression of thirty-six lncRNAs is closely associated with multiple clinical characteristics of BC, including tumor size, metastasis, invasion, and drug sensitivity or resistance of BC. Furthermore, we summarize some potential diagnostic and prognostic biomarkers of lncRNA for BC. This review provides promising novel biomarkers in early diagnosis, prognosis and monitoring of BC based on lncRNAs.

## Graphical Abstract

LncRNAs interacted with proteins in BC. **(A)** UCA1 interacts with C/EBPα. **(B)** Lnc-LBCS directly binds to hnRNPK and EZH2, and inhibits SOX2 transcription. **(C)** XIST-TET1-p53 pathway in BC. **(D)** DGCR5 promotes P21 transcription via interacting with ARID1A. **(E)** LSINCT5 promotes tumor progression by interacting with NCYM, and inhibiting GSK3β activity and promoting Wnt/β-catenin signaling activation. **(F)** GAS5 inhibits EZH2 transcription by interacting with E2F4. **(G)** AWPPH promotes cell proliferation, autophagy, and migration through binding to SMAD4 via EZH2. P indicates promoter. **(H)** LNMAT1 regulates CCL2 expression through interaction with hnRNPL. Protein-RNA interactions are important aspects of many cellular functions, and lncRNAs are involved in modulating BC through different molecular mechanisms, including binding to one or more protein partners.

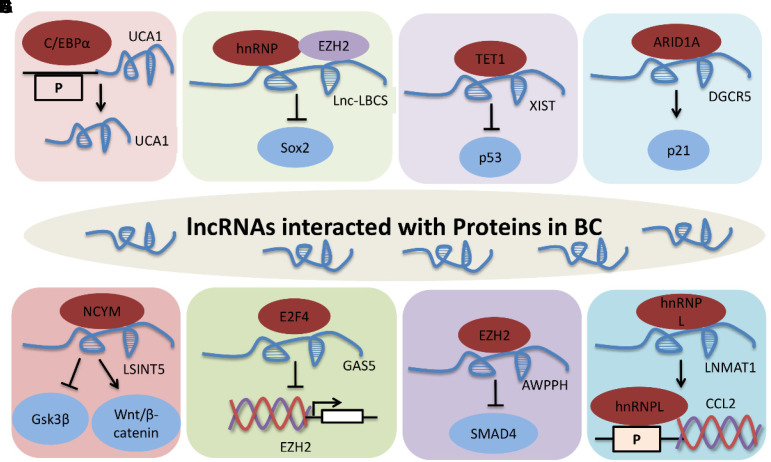

## Highlights

- The abnormal expression of 100 lncRNAs is involved in regulating of proliferation, cell cycle, apoptosis, migration, invasion, metabolism and drug resistance of bladder cancer.
- The abnormal expression of thirty-six lncRNAs is closely related to multiple clinical parameters, including tumor size, metastasis, invasion, tumor lymph node metastasis stage, diagnosis and prognosis of bladder cancer.
- LncRNAs regulate bladder cancer by interacting with DNA, RNA and proteins.
- This review will provide novel strategies and biomarkers for diagnosis, prognosis and monitoring of bladder cancer base on lncRNAs.

## Introduction

Bladder cancer (BC) is one of the most common malignant tumors in the urinary system, and its incidence is increasing. It is estimated that 81,400 new cases and 17,980 deaths of BC in the United States in 2020 (Siegel et al., 2020). Clinically, 75–80% of BC patients exhibit moderately differentiated non-muscle-invasive bladder cancer (NMIBC) at the initial diagnosis, and the remaining cases are muscle-invasive bladder cancer (MIBC). The standard treatment of NMIBC includes transurethral resection of bladder tumor (TUR-Bt), but high recurrence rate of BC after TUR-Bt and a subsequent tumor progression. The 5-year survival rate of patients with NMIBC is approximately 90%, while the 5-year survival rate of MIBC patients is only 20–40%, due to rapid disease progression and some early symptoms.

Long non-coding RNA (lncRNA) is defined as non-protein-coding RNA molecule longer than 200 nucleotides (Iyer et al., 2015). LncRNA plays an important regulatory role in epigenetic modification, transcription and post-transcriptional processing. Recent findings highlight that lncRNA contributes to the regulation of multiple signaling pathways in BC. LncRNA urethral cancer associated 1 (UCA1) was cloned from two human bladder transitional cell carcinoma cell lines. UCA1 is highly expressed in BC, and promotes progression, glycolysis, glutamine metabolism, migration and invasion of BC through the UCA1-mTOR/STAT3/miR-143/HK2 and UCA1/miR-16/GLS2 signaling network (Li et al., 2014, 2015). Increasing evidence suggests that lncRNA exert their roles during the biological processes of tumorigenesis, tumor proliferation, differentiation, apoptosis, invasion, migration and stemness (Zhang et al., 2013; Nicolas, 2017; Zhang and Tang, 2018).

In this review, we focused on the expression, function and related genes or signaling pathways of lncRNAs, as well as the molecular mechanisms of lncRNAs and their clinical significance for diagnosis and prognosis in BC, which can provide new strategies and novel biomarkers for the diagnosis and treatment of BC based on lncRNAs.

## Importance of Novel Biomarkers for BC

At present, diagnostic tests for BC include cystoscopy, urine cytology, FDA-approved markers, and non-FDA-approved markers. Four urinary FDA-approved commercial urinary biomarker tests are as follows: (1) bladder tumor antigen stat (BTA stat) and BTA TRAK; (2) Nuclear Matrix Protein 22 (NMP22) BC Test and NMP22 BladderChek; (3) uCyt+ assay; and (4) UroVysion assay. Several new biomarkers at the genomic, transcriptomic, and epigenetic levels have been developed, including: (1) ADXBLADDER, a novel ELISA test for Mini-chromosome maintenance 5; (2) Cxbladder Monitor; (3) XPERT BC, measure mRNA levels of ABL1, CRH, IGF2, UPK 1B, and ANXA 10; (4) UroMark assay; and (5) AssureMDX, test of the DNA methylation of OTX1, ONECUT2, and TWIST1 and somatic statuses of FGFR3, TERT, and HRAS in urine (Dangle et al., 2009; Tabayoyong and Kamat, 2018). Due to their poor sensitivity and specificity, cystoscopy and urine cytology are still used as the gold standard for the diagnosis of BC. Cystoscopy is the primary method for the diagnosis and monitoring of BC, and is not only invasive, entailing complications such as urinary tract infection or hematuria, but is also expensive (Casadevall et al., 2017). The urine cytology test is used to detect exfoliated malignant cells under the microscope, and is a non-invasive examination. It has high sensitivity and specificity for detection of high-grade BC; however, its sensitivity is only 4–31% for low-grade malignant tumor. In addition, false positive rate is as high as 12% due to inflammation and urothelial heterogeneity (Burchardt et al., 2000).

Early diagnosis of BC is essential for improvement of the prognosis and overall survival of patients. Given the current limited diagnostic methods, it is important to develop novel specific biomarkers as non-invasive tools that will facilitate accurate diagnosis and highly personalized treatment.

## The Definition, Features, and Classification of lncRNAs

Of the 3 billion base pairs in the human genome, only 1.5% are protein-coding sequences, and the rest are non-protein-coding sequences. There are many types of ncRNA, including short-stranded ncRNA and lncRNA. A greater proportion of lncRNA encoded by the human genome has tissue or cell type specificity, and is even related to specific stages of biological development. The definition of lncRNA was first proposed by Okazaki Y in 2002, mainly due to their discovery of a large number of lncRNA transcripts in large-scale sequencing studies of mouse cDNA libraries (Okazaki et al., 2002). LncRNA is defined as RNA longer than 200 nucleotides that not translated into proteins.

Anatomy of the global features of lncRNA suggests that lncRNA has some typical features, as follows: (1) lncRNA is usually larger than 200 nucleotides, (2) most of them have the eukaryotic structure of mRNA, the structure of promoter and polyA tail after RNA splicing, (3) contains few and long exons, (4) low expression, (5) less evolutionally conserved.

LncRNA has a variety of classifications according to its different characteristics. Currently, lncRNAs are mainly classified relying on four characteristics, including the location within the genome, the effect on the DNA sequence, the mechanisms of function, and targeting mechanisms of lncRNAs. According to the position of lncRNA in the genome and protein-coding genes, it is usually classified into five categories: sense lncRNA, antisense lncRNA, bidirectional lncRNA, intragenic lncRNA, and intergenic lncRNA. Based on the effect of lncRNA on DNA sequence, it can be divided into *cis*-lncRNAs and trans-lncRNAs. According to function mechanisms of lncRNA, it is fall into transcriptional regulation, post-transcriptional regulation, and translational regulation. Depending on the targeting mechanism of lncRNAs, it can be divided into signal, decoy, guide and scaffold (Ma et al., 2013).

## Expression and Role of lncRNAs in BC

To date, 120,353 human annotated lncRNAs have been collected by the LNCipedia database, and more than 70,000 published human and mouse lncRNAs have been annotated by NON-CODE (Zhang et al., 2013). In general, lncRNAs show more tissue-specific patterns and higher expression variability than protein-coding genes in cell lines and tissues. A large fraction of lncRNAs preferentially localize in the nucleus, some are located in the cytoplasm, and a few are found both in the cytoplasm and the nucleus (Ponting et al., 2009). Nuclear lncRNAs are involved in diverse functions, such as histone modifications, chromatin remodeling, modulation of gene expression *in cis* or *trans*. Cytoplasmic lncRNAs can perform cytoplasmic functions, including post-transcriptional modification of mRNAs, interaction with proteins and miRNAs sponging.

### Upregulated lncRNAs in BC

There are more than 100 dysregulated lncRNAs engaged in regulation of various biological functions of BC, such as cell proliferation, apoptosis, and metastasis. As shown in Table 1, forty-three upregulated lncRNAs involved in proliferation, migration, invasion, and cell cycle of BC, such as UCA1, long non-coding RNA 19 (H19), taurine up-regulated gene 1 (TUG1), and Calmodulin Like 3 Antisense RNA 1 (CALML3-AS1). Cytoplasmic lncRNAs can serve as oncogenes. UCA1, which is overexpressed in bladder cancer, specifically induces Glutaminase 2 (GLS2) by sponging miR-16, and can also activate AKT by recruiting E1A Binding Protein P300 (EP300) and lead to bladder cancer cell growth. UCA1 has a further oncogenic function by enhancing mTOR/STAT3/HK2 signaling pathway, which promotes Warburg effect of bladder cancer (Li et al., 2014). Additionally, UCA1 is modulated by upstream molecules, such as bone morphogenetic protein 9 (BMP9), which then promotes tumor progression of bladder cancer.

**TABLE 1:** Upregulated expression, function and related gene or signaling pathway of lncRNAs in BC.

LncRNA	Function	Pathways and genes	Samples	References
UCA1	P, M, I, C, A, G, Mit, CR	BMP9/pAKT/UCA1, miR-1/UCA1, UCA1/mTOR-STAT3/HK2, UCA1/CREB/miR-196a-5p, UCA1/BRG1/p21, C/EBPα/UCA1, UCA1/cell cycle related genes (CDKN2B, EP300 and TGFβ-2)	BC tissues, cell lines	Yang et al., 2012; Wang et al., 2014; Xue et al., 2014; Li et al., 2015; Li H. J. et al., 2017; Pan et al., 2016
H19	P, M, I, C, A	H19/miR-29b-3p/DNMT3B, H19/miR-675/p53	BC tissues, cell lines	Liu et al., 2016; Lv et al., 2017
TUG1		TUG1/HMGB1, TUG1/miR-29c, miR-142/ZEB2	BC tissues, cell lines	Jiang et al., 2017; Liu Q. et al., 2017; Guo et al., 2018
CALML3-AS1		CALML3-AS1/miR-4316/ZBTB2	BC tissues, cell lines	Wang et al., 2018a
NEAT1		NEAT1/miR-410/HMGB1	Cell lines	Shan et al., 2020a
Lnc-MUC20-9	P, M, I, A	ROCK1	Cell lines	Dai et al., 2020
lncRNA-SNHG20		lncRNA-SNHG20/Wnt/β-catenin	BC tissues, cell lines	Zhao et al., 2018
HOTAIR		HuR/HOTAIR/miR-1, HOTAIR/miR-205/Cyclin J	BC tissues, cell lines	Sun et al., 2015; Chen et al., 2017b; Yu D. et al., 2017
SPRY4-IT1		SPRY4-IT1/miR-101-3p/EZH2	BC tissues, cell lines	Liu D. et al., 2017
SNHG16		SNHG16/miR-98/STAT3/Wnt/β-catenin, SNHG16/P21	BC tissues, cell lines	Cao et al., 2018; Feng F. et al., 2018
ZEB1-AS1		ZEB1-AS1/miR-200b/FSCN1	Cell lines	Gao et al., 2019
KCNQ1OT1		miR-145-5p/PCBP2	Cell lines	Fang et al., 2019b
PVT1		PVT1/miR-218/VEGFC, PVT1/Wnt/β-catenin	BC tissues, cell lines	Liu and Zhang, 2017; Yu C. et al., 2019
NNT-AS1		NNT-AS1/miR-1301-3p/PODXL/Wnt,	Cell lines	Liu and Wu, 2020
LncARSR	P, M, I, EMT	LncARSR/miR-129-5p/SOX4	BC tissues, cell lines	Liao et al., 2020
DLX6-AS1		DLX6-AS1/miR-223/HSP90B	BC tissues, cell lines	Fang et al., 2019b
MNX1-AS1		miR-218-5p/RAB1A	cell lines	Wang et al., 2020
lncRNA ROR	P, M, A, C	lncRNA ROR/ZEB1	BC tissues, cell lines	Chen et al., 2017a
AWPPH	P, M, I	AWPPH/EZH2/SMAD4	BC tissues, cell lines	Zhu F. et al., 2018
linc-UBC1		linc-UBC1/EZH2/SUZ12	BC tissues, cell lines	He et al., 2013
MAGI2-AS3		MAGI2-AS3/miR-15b-5p/CCDC19	BC tissues, cell lines	Wang et al., 2018b
SLCO4A1-AS1		SLCO4A1-AS1/miR-335-5p/OCT4	BC tissues, cell lines	Yang et al., 2019
PEG10		PEG10/miR-134/LRP6	BC tissues, cell lines	Jiang et al., 2019
LSINCT5	M, I	LSINCT5/NCYM/GSK3β/Wnt/β-catenin	Cell lines	Zhu X. et al., 2018
AATBC	P, C, A	AATBC/(cyclinD1, CDK4, p18, Rb)	BC tissues, cell lines	Zhao et al., 2015
BCAR4		BCAR4/miR-370-3p/Wnt7a	BC tissues, cell lines	Zhang et al., 2020
DUXAP10		DUXAP10/(Bcl-xL, cyclin D, CDK4, Bad, caspase3/9, p27, PI3K/Akt/mTOR)	Cell lines	Lv et al., 2018
DUXAP8	P	DUXAP8/PTEN	BC tissues, cell lines	Lin et al., 2018
PTENP1		PTENP1/miR-17/PTEN	BC tissues, cell lines	Yu G. et al., 2017
NORAD		NORAD/(PUM2, E2F3)	BC tissues, cell lines	Li Q. et al., 2018
SNHG5		SNHG5/P27	BC tissues, cell lines	Ma et al., 2018
ARAP1-AS1		ARAP1-AS1/miR-4735-3p/NOTCH2	BC tissues, cell lines	Teng et al., 2019
CASC11		CASC11/miR-150	Plasma, cell lines	Luo et al., 2019
ITGB1		ITGB1/miR-10a	BC tissues, cell lines	Dai et al., 2019
linc00511		linc00511/Wnt/β-catenin	BC tissues, cell lines	Li J. et al., 2018
SOX2OT	P, A	SOX2OT/SOX2	BC tissues, cell lines	Zhan et al., 2020
OXCT1-AS1	P, M, S	OXCT1-AS1/miR-455-5p/JAK1	BC tissues, cell lines	Chen J. B. et al., 2019
XIST	P, M, A	XIST/(TET1, p53)	Cell lines	Hu et al., 2019
CASC8	P, I	CASC8/FGFR1/LDHA	BC tissues, cell lines	Hu et al., 2017
LINC01638	P, G	LINC01638/ROCK2	Plasma, cell lines	Yuan et al., 2019
LNMAT1	EMT	LNMAT1/hnRNPL/CCL2/VEGF-C	BC tissues, cell lines	Chen et al., 2018
LNMAT2	Lymphatic metastasis	LNMAT2/hnRNPA2B1/PROX1	BC tissues, cell lines	Chen C. et al., 2020
MST1P2	Prognostic factor	MST1P2/miR-133b/Sirt1/p53	Cell lines	Chen J. et al., 2020

**P, proliferation; M, migration; I, invasion; A, apoptosis; Mit, mitochondria; AP, autophagy; C, Cell cycle; G, glycolysis; D, differentiation; Me, methylation; S, Stemness; CR, Cisplatin resistance. UCA1, urothelial cancer associated 1; TUG1, taurine up-regulated gene 1; DLEU1, deleted in lymphocytic leukemia 1; HOTAIR, HOX transcript antisense RNA; SPRY4-IT1, Sprouty4 intronic transcript 1; SNHG16, Small nucleolar RNA host gene 16; ZEB1-AS1, Zinc finger E-box binding homeobox 1 antisense 1; KCNQ1OT1, KCNQ1 overlapping transcript 1.**

LncRNAs can also be oncogene by regulating signaling molecules in the nucleus. Linc-UBC1 is upregulated and preferentially localizes in nucleus, which aggravates BC progression through altering polycomb repressive complex 2 (PRC2) complex localization by interacting with enhancer of zeste 2 polycomb repressive complex 2 subunit (EZH2) and SUZ12 polycomb repressive complex 2 subunit (SUZ12) (He et al., 2016). Similar to lncRNA upregulated in bladder cancer 1 (linc-UBC1), associated with poor prognosis of hepatocellular carcinoma (AWPPH) and non-coding RNA activated by DNA damage (NORAD) play regulatory roles in nucleus of bladder cancer cells.

A few lncRNAs can act as both cytoplasmic lncRNA and nuclear lncRNA. H19 promoter region can bind to CCCTC-binding factor (CTCF) and insulin like growth factor 2 (IGF2), and thereby regulating bladder cancer development in nucleus of BC cells (Takai et al., 2001). It was also reported that H19 promotes epithelial-mesenchymal transition and metastasis of bladder cancer via H19/miR-29b/DNMT3B signaling pathway in cytoplasm (Lv et al., 2017).

### Downregulated lncRNAs in BC

As shown in Table 2, seventeen downregulated lncRNAs involved in regulation of BC. Among them, MIR503 host gene (MIR503HG), LncRNA maternally expressed gene 3 (MEG3), lncRNA miR143 host gene (MIR5143HG), lncRNA cancer susceptibility candidate 2 (CASC2) (Pei et al., 2017), phosphatase and tensin homolog pseudogene 1 (PTENP1) (Zheng et al., 2018) and BRAF-activated non-coding RNA (BANCR) (He et al., 2016) are involved in regulating cell proliferation, migration and apoptosis. In addition, MIR503HG is engaged in the regulation of cell invasion and cycle, MEG3 is involved in regulating cell invasion, cycle and autophagy, lncRNA miR143HG regulates cell cycle, and CASC2 and PTENP1 medicate cell invasion. LINC00312 and LINC00641 are participated in regulating cell proliferation, migration and invasion (Li Z. et al., 2018). LOC572558 and NBAT1 are involved in modulating proliferation, migration and cell cycle (Zhu et al., 2016; Liu and Zhang, 2017; Liu Z. et al., 2018). The nuclear lncRNA deleted in bladder cancer chromosome region 1-003 (DBCCR1-003) can suppress DBCCR1 transcription by methylation of promoter region of DBCCR1 in T24 cells. The cytoplasmic lncRNA MEG3 inhibits bladder cancer progression by regulating multiple signaling axis, such as miR-96/TPM1, miR-27a/PHLPP2 and miR-494/PTEN.

**TABLE 2:** Downregulated expression, function and related gene or signaling pathway of lncRNAs in BC.

Name	Expression in BC	Function	Pathways and genes	Samples	References
MIR503HG	Down	P, M, I, C, A	MIR503HG/(ZEB1, Snail, N-cadherin, and vimentin, E-cadherin)	BC tissues, cell lines	Qiu et al., 2019
MEG3	Down	P, M, I, C, A, AP	MEG3/miR-96/TPM1, MEG3/miR-494/PTEN, MEG3/LC3-I/II, MEG3/miR-27a/PHLPP2	BC tissues, cell lines	Ying et al., 2013; Feng S. Q. et al., 2018; Liu G. et al., 2018; Shan et al., 2020b
lncRNA miR143HG	Down	P, M, A, C	miR143HG/miR-1275/AXIN2	BC tissues, cell lines	Xie et al., 2019
CASC2	Down	P, M, I, A	CASC2/Wnt/β-catenin	BC tissues, cell lines	Pei et al., 2017
PTENP1	Down	P, M, I, A	PTENP1/miR-17/PTEN	BC tissues, cell lines	Zheng et al., 2018
BANCR	Down	P, M, A	-	BC tissues, cell lines	He et al., 2016
LINC00312	Down	P, M, I	LINC00312/miR-197-3p, MMP2/9/TIMP2	BC tissues, cell lines	Wang et al., 2016
LINC00641	Down	P, M, I	LINC00641/miR-197-3p/KLF10/PTEN/PI3K/AKT	BC tissues, cell lines	Li Z. et al., 2018
LOC572558	Down	P, M, C	LOC572558/AKT/MDM2/p53	BC tissues, cell lines	Zhu et al., 2016
NBAT1	Down	P, M, C	NBAT1/miR-21/SOCS6	BC tissues, cell lines	Liu Z. et al., 2018
MT1JP	Down	P, I, C	MT1JP/miR-214-3p	BC tissues, cell lines	Yu H. et al., 2019
DGCR5	Down	P, C	DGCR5/ARID1A/P21	BC tissues, cell lines	Fang et al., 2019a
GAS5	Down	P, A	GAS5/miR21/PTEN, GAS5/(CCL1, Bcl2, CDK6)	BC tissues, cell lines	Cao et al., 2016; Chen D. et al., 2020
MBNL1-AS1	Down	P, A	MBNL1-AS1/miR-135a/PHLPP2/FOXO1	BC tissues, cell lines	Wei et al., 2020
TP73-AS1	Down	P	TP73-AS1/(vimentin, MMP-2/9, snail, E-cadherin)	BC tissues, cell lines	Tuo et al., 2018
lncRNA-LBCS	Down	P	hnRNPK, EZH2, SOX2, H3K27	BC tissues, cell lines	Chen X. et al., 2019
DBCCR1-003	Down	Me	DBCCR1-003/DNMT1/methylation of DBCCR1	BC tissues, cell lines	Qi et al., 2016

**P, proliferation; M, migration; I, invasion; A, apoptosis; AP, autophagy; C, Cell cycle; Me, methylation.**

## Functional Mechanisms of lncRNAs on BC

Gene expression is modulated by lncRNAs at various levels. The mechanistic characterization of lncRNAs on bladder cancer is not well documented. It is partly due to the fact that lncRNAs harbor more cell type specificity and tissue type specificity, and generally low expression level. In spite of lncRNAs are less conversed among different species, there are similarities in functional mechanisms in bladder cancer. In this section, we highlight uncover molecular mechanisms from the following three aspects: lncRNAs by interacting with DNA, RNA and proteins.

### LncRNAs Affect BC by Interacting With DNA

**FIGURE 1:**
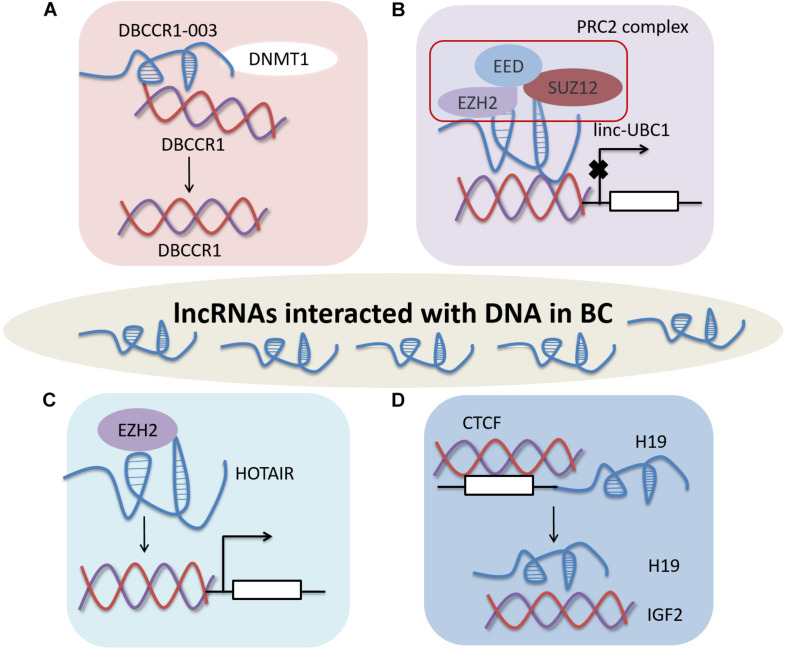
LncRNAs affect BC by interacting with DNA. **(A)** DBCCR1-003 regulates the expression of DBCCR1 by binding to DNMT1 and inhibiting DNMT1-mediated methylation of DBCCR1. **(B)** Linc-UBC1 modulating the PRC2 complex by combining with EZH2 and SUZ12. **(C)** HOTAIR modulates gene expression by binding to EZH2. **(D)** CTCF regulates expression of H19 by methylating the H19 promoter in human BC.

LncRNAs may anchor to promoters or regulators of target DNA to regulate gene expression in BC (Figure 1). LncRNA DBCCR1-003 as a tumor suppressor, derived from the DBCCR1 locus. DBCCR1-003 modulates the expression of DBCCR1 via DNA methylation in bladder cancer tissues and T24 cell lines. DBCCR1-003 can bind to DNA methyltransferase 1 (DNMT1), but the expression of DNMT1 remains unchanged when DBCCR1-003 is overexpressed in T24 cells. The results reveal that lncRNA DBCCR1-003 can modulate the expression of DBCCR1 by binding to DNMT1 and inhibiting DNMT1-mediated methylation of DBCCR1 in BC (Qi et al., 2016).

Linc-UBC1 can physically bind to EZH2 and SUZ12, key component of PRC2 complex. Some PRC2 target genes, such as Cyclin D2 (CCND2), plasminogen activator inhibitor type 2 (SERPINB2), Bone morphogenetic protein 2 (BMP2), Kruppel-like factor 4 (KLF4) and Homeobox A5 (HOXA5), are downregulated after knockdown of linc-UBC1. Moreover, H3K27 trimethylation in the promoter regions of CCND2, SERPINB2, the expression levels of BMP2 and HOXA5 decreased with the low expression of linc-UBC1. These results indicate that linc-UBC1 may partly modulate PRC2 complex localization by regulating histone H3K27 trimethylation with EZH2 and SUZ12 (He et al., 2013).

LncRNA HOX transcript antisense intergenic RNA (HOTAIR) modulates gene expression by binding to enzymes, such as EZH2, to inhibit histone modifications. HOTAIR mostly depends on EZH2, which is significantly increased in BC. HOTAIR originates from the HOX locus, which regulates HOX gene expression, differentiation and tissue homeostasis (Heubach et al., 2015). Additionally, the expression of HOTAIR is regulated by EZH2 after reduction of EZH2 in 5637 cells (Mónica et al., 2015).

Human H19 promoter contains large amount of methylcytosine in the CTCF binding site of and is abnormal hypomethylated in human bladder cancer. The sixth CTCF-binding site may serve as a significant regulatory domain, thereby switching H19 or IGF2 expression in human bladder cancer (Takai et al., 2001). Up to now, only a small number of lncRNAs has been demonstrated to participate in the regulation of BC through interacting with DNA.

### LncRNAs Affect BC Through Interacting With RNA

**FIGURE 2:**
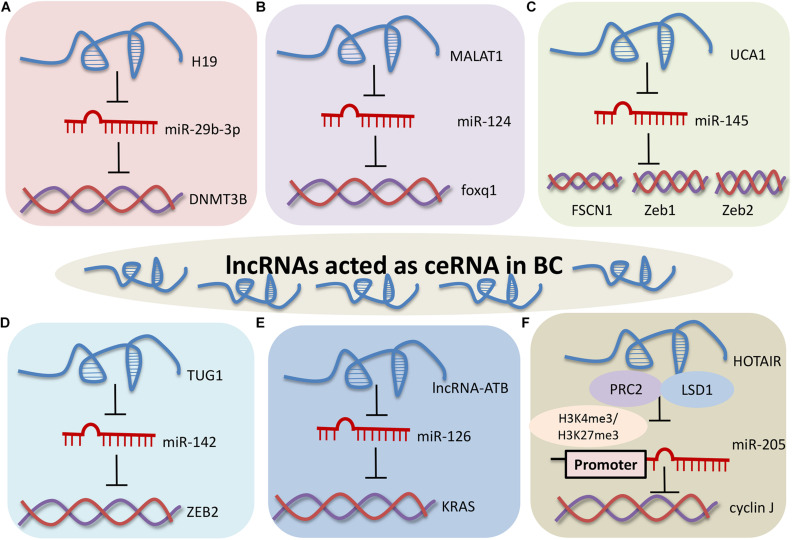
LncRNAs exert their biological effects by sponging miRNAs in BC. **(A)** H19 regulated EMT and metastasis of BC through the H19/miR-29b-3p/DNMT3B axis. **(B)** MALAT1 promoted development of BC through the MALAT1/miR-124/foxq1 signaling pathway. **(C)** UCA1 enhanced BC migration and invasion through the UCA1-miR-145/FSCN1/Zeb1/Zeb2 network. **(D)** TUG1 enhanced proliferation of BC through the TUG1/miR-142/ZEB2 axis. **(E)** LncRNA-ATB positively regulated BC through the lncRNA-ATB-miR-126/KRAS. **(F)** The HOTAIR/miR-205/cyclin J signaling pathway in BC.

Accumulating data indicate that both coding and non-coding RNAs can regulate one another by competing miRNA binding, known as competing endogenous RNAs (ceRNAs) (Salmena et al., 2011). ceRNAs can decoy miRNAs and protect their target mRNAs from degradation (Kartha and Subramanian, 2014). According to the ceRNA hypothesis, lncRNAs may exert their biological effects in BC by sponging miRNAs, which would in turn modulate the effect of miRNAs on their targets (Figure 2).

H19 regulates the expression level of DNMT3B protein and epithelial-mesenchymal transition in bladder cancer. H19 can directly combine with the seed region of miR-29b-3p to sequester H19 by dual luciferase reporter assay, FISH and RIP assay. The expression of DNMT3B is repressed by miR-29b-3p in T24 cells. Collectively, H19 regulates the epithelial-mesenchymal transition and metastasis of BC through the H19/miR-29b-3p/DNMT3B axis (Lv et al., 2017). There is significant negative correlation between metastasis-associated lung adenocarcinoma transcript 1 (MALAT1) and miR-124 in bladder transitional cell carcinoma tissues and normal mucosa samples. Foxq1, putative target gene of miR-124, is positively correlated with the expression of MALAT1 in bladder transitional cell carcinoma. Upregulation of MALAT1 promotes proliferation, migration, and invasion of bladder transitional cell carcinoma through the MALAT1/miR-124/foxq1 signaling pathway (Jiao et al., 2018). The exons 2 and 3 of UCA1 harbor miR-145 binding site and can suppress the expression of miR-145. ZEB1/2 and FSCN1 are engaged in migration and invasion of bladder cancer, and are the direct target genes of miR-145. These data suggest that UCA1 enhances BC migration and invasion through the miR-145-ZEB1/2-FSCN1 network (Xue et al., 2016). TUG1 and ZEB2 act as oncogene in bladder cancer. TUG1 contains the binding sites of miR-142, and TUG1 inhibits miR-142 expression in T24 and BIU-87 cells. These results imply that TUG1 enhances the proliferation and inhibits apoptosis of BC cells via the TUG1/miR-142/ZEB2 axis (Liu Q. et al., 2017). LncRNA-activated by transforming growth factor-β (lncRNA-ATB) can promote cell proliferation, migration and invasion by capturing miR-126 in T24 cells. KRAS is a direct target gene of miR-126 and is inversed regulated by miR-126. These results indicate that lncRNA-ATB promotes cell proliferation, invasion and migration by decoying miR-126 in bladder cancer (Zhai and Xu, 2018).

Unlike sponge miRNAs, HOTAIR can repress miR-205 transcription by recruiting PRC2 and lysine-specific demethylase 1 (LSD1), and then recruiting H3K4me3 and H3K27me3 in miR-205 promoter region in HCV29 cells. Cyclin J is a putative downstream target gene of miR-205 in bladder cancer. These findings suggest that HOTAIR participates in the regulation of proliferation, migration and invasion of bladder tumor cells through the HOTAIR/miR-205/cyclin J signaling pathway (Sun et al., 2015).

### LncRNAs Affect BC by Interacting With Proteins

**FIGURE 3:**
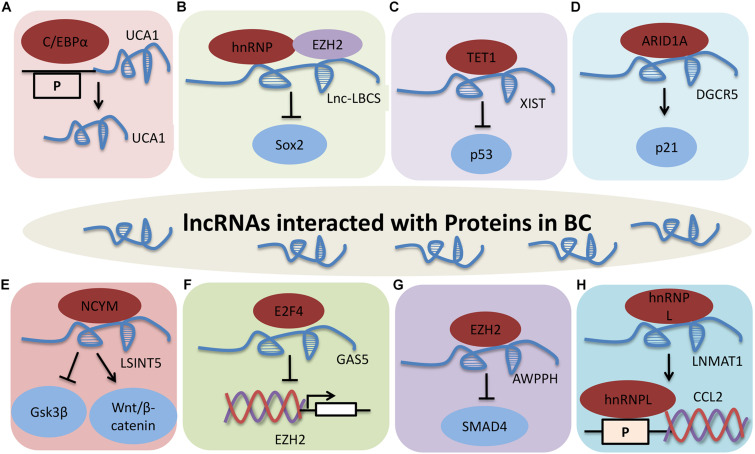
LncRNAs that interact with proteins in BC. **(A)** UCA1 interacts with C/EBPα. **(B)** Lnc-LBCS directly binds to hnRNPK and EZH2, and inhibits SOX2 transcription. **(C)** XIST-TET1-p53 pathway in BC. **(D)** DGCR5 promotes P21 transcription by interacting with ARID1A. **(E)** LSINCT5 promotes tumor progression by interacting with NCYM, and inhibiting GSK3β activity and promoting Wnt/β-catenin signaling activation. **(F)** GAS5 inhibits EZH2 transcription by interacting with E2F4. **(G)** AWPPH promotes cell proliferation, autophagy, and migration through binding to SMAD4 via EZH2. P indicates the promoter. **(H)** LNMAT1 regulates CCL2 expression through interacting with hnRNPL.

Protein-RNA interactions are important aspects of many cellular functions, and lncRNAs are involved in modulating BC through different molecular mechanisms, including binding to one or more protein partners (Figure 3). UCA1 is upregulated by C/EBPα through the combination of core promoter region of UCA1 with C/EBPα in BLZ-211, 5637 and T24 cells. C/EBPα enhances UCA1 transcription was detected by qRT-PCR and luciferase activity. These results indicate that UCA1 increases cell viability and inhibits cell apoptosis through the UCA1 core promoter interacting with C/EBPα (Xue et al., 2014).

LncRNA low expressed in Bladder Cancer Stem cells (Lnc-LBCS) is markedly downregulated in bladder cancer tissues, and inhibits the self-renewal, tumor initiation, and tumor sphere formation of BC. Lnc-LBCS can act as a scaffold to activate the formation of hnRNPK-EZH2 complex by directly binding to heterogeneous nuclear ribonucleoprotein K (hnRNPK) and EZH2. Lnc-LBCS medicates SOX2 expression by forming hnRNPK-EZH2-SOX2 complex triplexes. Collectively, lnc-LBCS directly binds to hnRNPK and EZH2 and acts as a scaffold to induce complex formation to inhibit SOX2 transcription by regulating H3K27me3 (Chen X. et al., 2019).

LncRNA X-inactive specific transcript (XIST) facilitates cell growth and supresses cell apoptosis by reducing p53 expression in T24 and 5637 cells. XIST can bind to ten-eleven translocation 1 (TET1), and TET1 can enhance p53 expression by combing with the promoter region of p53. These data show that XIST regulates cell proliferation, migration and apoptosis via the XIST/TET1/p53 pathway in BC (Hu et al., 2019).

LncRNA DiGeorge syndrome critical region gene 5 (DGCR5) is significantly downregulated in bladder cancer. Overexpression of DGCR5 represses cell proliferation, cell cycle progression, migration, invasion, and EMT while promotes cell apoptosis of bladder cancer. DGCR5 is require for AT-rich interaction domain 1A (ARID1A)-induced P21 expression in T24 and SW780 cells. This study demonstrate that DGCR5 promotes P21 transcription by interacting with ARID1A in BC (Fang et al., 2019a).

Long stress-induced non-coding transcript 5 (LSINCT5) directly interacts with NCYM, and inhibits GSK3β activity, leading to Wnt/β-catenin signaling activation and promoting EMT in bladder cancer cell lines (Zhu X. et al., 2018). High GAS5 expression is correlated with poor prognosis in BC, and GAS5 effectively inhibits EZH2 transcription by binding and recruiting E2F4 to EZH2 promoter region, to promote BC cell apoptosis (Wang et al., 2018c). LncRNA AWPPH is highly expressed in BC and induces cell proliferation, autophagy, and migration, and restrains cell apoptosis by suppressing SMAD4 and EZH2 (Zhu F. et al., 2018).

Long non-coding RNA lymph node metastasis associated transcript 1 (LNMAT1) promotes bladder cancer cells to lymph node and lymphangiogenesis *in vivo*. Chemokine (C-C motif) ligand 2 (CCL2) is necessary for LNMAT1-mediated lymph node metastasis, and is regulated by the LNMAT1-CCL2 promoter through directly interacting with hnRNPL in UM-UC-3 cells. Taken together, LNMAT1 regulates CCL2 expression through physically interaction with hnRNPL in BC (Chen et al., 2018).

## LncRNAs Could Be Promising Diagnostic or Prognostic Biomarker in BC

In recent years, accumulating evidence has demonstrated that abnormal expression of lncRNAs has crucial clinical significance for the diagnosis of BC, and is closely related to multiple clinical characteristics of BC, including tumor size, metastasis, invasion, tumor node metastasis (TNM) stage, diagnosis and prognosis (Table 3).

**TABLE 3:** Correlation of clinical parameters and lncRNAs in BC.

Clinical parameters	Upregulated lncRNAs	Downregulated lncRNAs	Number
Tumor size	DUXAP8, GAPLINC, ZFAS1, NORAD, SNHG5, SNHG16, PCAT-1, TUG1, MALAT1, CAT266, CAT1297, CAT1647, linc-UBC1, UCA1, LSINCT5	MEG3, TP73-AS1, PTENP1	18
Metastasis/Invasion/TNM stage	FGFR3-AS1, DUXAP8, CRNDE, ZFAS1, NORAD, MALAT1, XIST, CASC8, CCEPR, ZEB1-AS1, ABHD11-AS1, PANDAR, CCAT2, SUMO1P3, UCA1	CASC2, GAS5, BANCR	18
Drug sensitivity/Resistance	CDKN2B-AS, PVT1, UCA1, HOTAIR, SNHG16, Linc00857, FOXD2-AS1	GAS5	8
Radiosensitivity	TUG1		1
Total	30	6	

As shown in Table 3, eighteen lncRNAs are related to tumor size, including double homeobox A pseudogene 8 (DUXAP8), gastric adenocarcinoma predictive long intergenic non-coding RNA (GAPLINC), zinc finger antisense 1 (ZFAS1), P73 antisense RNA 1?T (TP73-AS1), non-coding RNA-activated by DNA damage (NORAD), small nucleolar RNA host gene 5 (SNHG5), small nucleolar RNA host gene 16 (SNHG16), prostate cancer associated transcript-1 (PCAT-1), TUG1, MALAT1, CAT266, CAT1297, CAT1647, linc-UBC1, UCA1, MEG-3, LSINCT5, and PTENP1. 18 lncRNAs are implicated in metastasis, invasion and TNM stage, including FGFR3 antisense transcript 1 (FGFR3-AS1), DUXAP8, lncRNA colorectal neoplasia differentially expressed (lncRNA CRNDE), ZFAS1, NORAD, MALAT1, XIST, cancer susceptibility candidate 8 (CASC8), cervical carcinoma expressed PCNA regulatory lncRNA (CCEPR), zinc finger E-box binding homeobox 1 antisense 1 (ZEB1-AS1), CASC2, ABHD11 antisense RNA 1 (ABHD11-AS1), growth arrest specific transcript 5 (GAS5), BANCR, p21-associated ncRNA DNA damage-activated (PANDA), CCAT2, small ubiquitin-like modifier 1 pseudogene 3 (SUMO1P3) and UCA1. There are 8 lncRNAs associated with drug sensitivity/resistance, including CDKN2B antisense RNA 1 (CDKN2B-AS), lncRNA plasmacytoma variant translocation 1 (lncRNA PVT1), GAS5, UCA1, HOTAIR, SNHG16, Linc00857, and forkhead box D2 antisense 1 (FOXD2-AS1). Only lncRNA TUG1 contributes to radiosensitivity in bladder cancer.

In view of low expression, cell-specificity and tissue-specificity, lncRNAs represent favorable features to act as diagnostic or prognostic indicators for BC. Considering the low expression of lncRNA, the upregulated lncRNAs are more conducive to diagnosis for bladder cancer. Therefore, we selected potential clinical biomarkers among 30 up-regulated lncRNAs.

There are fifteen upregulated lncRNAs that contribute to tumor size of bladder cancer, including DUXAP8, GAPLINC, ZFAS1, NORAD, SNHG5, SNHG16, PCAT-1, TUG1, MALAT1, CAT266, CAT1297, CAT1647, linc-UBC1, UCA1 and LSINCT5. As shown in Supplementary Table 1, only three upregulated lncRNAs of MALAT1, SNHG16 and UCA1 have complete diagnostic data. The sensitivity of MALAT1, SNHG16 and UCA1 is 0.567, 0.642, 0.83; the specificity of MALAT1, SNHG16 and UCA1 is 0.675, 0.650, 0.86; the ROC curve AUC of MALAT1, SNHG16 and UCA1 used for bladder cancer is 0.635, 0.679, 0.86 (Duan et al., 2016; Ding et al., 2021). Taken together, UCA1 has relatively high sensitivity, specificity and AUC, and it can be regarded as the most potential diagnostic biomarker for bladder cancer.

There are fifteen upregulated lncRNAs significantly associated with over survival of BC patients, including FGFR3-AS1, DUXAP8, CRNDE, ZFAS1, NORAD, MALAT1, XIST, CASC8, CCEPR, ZEB1-AS1, ABHD11-AS1, PANDAR, CCAT2, SUMO1P3, and UCA1. As reported by Su et al. (2018) only MALAT1 is an independent prognostic biomarker for bladder cancer, suggesting that MALAT1 can be considered as a possible prognostic indicator of BC.

As described above, it should be considered that lncRNAs can serve as feasible diagnostic and prognostic biomarkers. However, new lncRNA biomarkers used for clinical diagnosis must previously be carried out extensive studies in cells and clinical specimen. A single lncRNA may not be a reliable indicator for accurate and timely detection of bladder cancer. In this regard, additional systematic studies on lncRNAs will facilitate the diagnostic and therapeutic performance for bladder cancer.

As the current literature on lncRNA and bladder cancer pays more attention to the correlation between lncRNA and bladder cancer, there is still no certain lncRNA for clinical diagnosis, prognosis and subsequent therapy of bladder cancer. It help to combination of traditional indicators with novel lncRNA biomarkers for diagnosis and prognosis of bladder cancer.

## Discussion

Over the past decade, accumulating findings have uncovered that lncRNAs are crucial for initiation and progression of bladder cancer. Up to now, standard bladder cancer biomarkers are still very rare, owing to lack of the high sensitivity and specificity, and high costs of use. Due to the high recurrence and poor prognosis of bladder cancer, even after effective transurethral resection and systemic treatment, it is require to develop novel biomarkers for the early diagnosis and prognosis of bladder cancer. In this review, we summarize the expression, function, molecular mechanisms of lncRNAs, and the clinical significance of lncRNAs in the diagnosis and prognosis of bladder cancer. The molecular mechanisms of lncRNAs in bladder cancer have been explored, including lncRNAs interacting with DNA, RNA and proteins.

Circulating lncRNAs can be enriched in urine supernatant and plasma of bladder cancer, which may have a preferable potential for new bladder cancer tests. It has been suggested that aberrant expression of thirty-six lncRNAs is closely related to multiple clinical characteristics of bladder cancer. In the light of low expression, less evolutionally conserved, the upregulated lncRNAs represent favorable features to act as diagnostic or prognostic indicators for BC. Therefore, we screened potential clinical biomarkers among thirty up-regulated lncRNAs. Dissection of fifteen up-regulated lncRNAs associated with tumor size of bladder cancer, UCA1 has relatively high sensitivity, specificity and AUC, and it can be considered as the most potential diagnostic biomarker for bladder cancer.

Importantly, novel lncRNA biomarkers for clinical diagnosis must previously be carried out large studies in cells and clinical specimen. In this regard, extensive systematic studies on lncRNAs will facilitate the diagnostic and therapeutic performance for bladder cancer. It is worth noting that there is currently no any lncRNA can be applied to the specific diagnosis, prognosis and treatment of bladder cancer. In addition to mRNAs, microRNAs, circular RNAs and exosomes also play important roles in the development of BC. Our present knowledge indicates that combination of mRNAs, microRNAs and lncRNAs would hopefully better for improvement of early diagnosis and prognosis in bladder cancer.

## Data Availability Statement

The original contributions presented in the study are included in the article/Supplementary Material, further inquiries can be directed to the corresponding author/s.

## Author Contributions

H-JL, XG, and Z-KL were responsible for the framework and the writing of manuscript, and analysis of references. WQ, C-XH, and LX were mainly dedicated to searching and sorting of references. XZ and DZ were focused on the reference analysis. H-LC was mainly responsible for the revision and polishing of the manuscript. All authors have made great contributions.

## Conflict of Interest

The authors declare that the research was conducted in the absence of any commercial or financial relationships that could be construed as a potential conflict of interest.

## Publisher’s Note

All claims expressed in this article are solely those of the authors and do not necessarily represent those of their affiliated organizations, or those of the publisher, the editors and the reviewers. Any product that may be evaluated in this article, or claim that may be made by its manufacturer, is not guaranteed or endorsed by the publisher.
