# Supplementary material for: Role of Long Non-coding RNAs on Bladder Cancer
Source: Front Cell Dev Biol. 2021 Aug 4;9:672679. doi: 10.3389/fcell.2021.672679 (PMC8371405; doi:10.3389/fcell.2021.672679)
Supplement: Supplementary file 1 [file Table_1.docx]

**Table S1. The upregulated lncRNAs acted as diagnostic biomarkers of BC.**

| **lncRNAs** | **Patient population (Sample size)** | |  |  |  |  |  |  |
| --- | --- | --- | --- | --- | --- | --- | --- | --- |
|  | **Cases** | **Controls** | **Sensitivity** | **Specificity** | **AUC** | **Sample** | **Detetion method** | **Ref** |
| DUXAP8 | 31 BC tissues | 31adjacent tissues | - | - | - | tissues | qRT-PCR | Lin 2018  China, Guangdong |
| GAPLINC | 80 BC tissues | 80 adjacent tissues | - | - | - | tissues | qRT-PCR | Zheng 2018  China, Guangdong |
| ZFAS1 | 86 BC tissues | 86 adjacent tissues | - | - | 0.8743 | tissues | qRT-PCR | Wang 2017  China, Shanghai |
| NORAD | 10 BC tissues | 10 adjacent tissues | - | - | - | tissues | qRT-PCR | Li 2018  China, Hunan |
| SNHG5 | 67 BC tissues | 67 adjacent tissues | - | - | - | tissues | qRT-PCR | Ma 2018  China, Shandong |
| MALAT1 | 100 BC tissues | 48 healthy subjects, 52 benign disease (21BPH, 15 Urolithiasis, 16 Cystitis) | 0.567 | 0.675 | 0.635 | tissue, serum and urine samples | qRT-PCR | Duan 2016 China, Shandong |
| SNHG16 | 100 BC tissues | 48 healthy subjects, 52 benign disease (21BPH, 15 Urolithiasis, 16 Cystitis) | 0.642 | 0.650 | 0.679 | tissue, serum and urine samples | qRT-PCR | Duan 2016  China, Shandong |
| SNHG16 | 26 BC tissues | 15 healthy controls | - | - | - | tissues |  | Feng 2018  China, Shandong |
| SNHG16 | 46 BC tissues | 46 adjacent tissues | - | - | - | tissues |  | Cao 2017  China, Shandong |
| SNHG16 | 275 bladder cancer patients |  | - | - | - | tissues | qRT-PCR | Peng 2018  China, Shandong |
| SNHG16 | 80 BC tissues | 80 adjacent tissues | - | - | - | tissues | qRT-PCR | Chen 2020  China, Fujian |
| SNHG16 | 260 BC tissues | 260 adjacent tissues | - | - | 0.681 | serum samples | qRT-PCR | Zhang 2018  China, Shandong |
| PCAT-1 | 260 BC tissues | 260 adjacent tissues | - | - | 0.753 | serum samples | qRT-PCR | Zhang 2018  China, Shandong |
| UBC1 | 260 BC tissues | 260 adjacent tissues | - | - | 0.751 | serum samples | qRT-PCR | Zhang 2018  China, Shandong |
| TUG1 | 22 BC tissues | 22 adjacent tissues | - | - | - | tissues | qRT-PCR | Guo 2018  China, Anhui |
| TUG1 | 50 BC tissues | 30 normal individuals | - | - | - | tissues | qRT-PCR | Abdolmaleki 2020 Iran |
| CAT266/ CAT1297/ CAT1647 | 122 MIBC tissue samples | 16 normal urothelium |  |  |  | TCGA project |  | Dudek 2017  The Netherlands |
| LSINCT5 | 108 BC tissues | 108 adjacent tissues | - | - | - | tissues | qRT-PCR | Zhu 2018  China, Henan |
| UCA1 | 954 bladder cancer patients | 482 non-bladder cancer patients | 0.83 | 0.86 | 0.86 | Meta Analysis |  | Ding 2021  China, Beijing |
